# Evaluation of *Streptomyces sporoverrucosus* B-1662 for biological control of red pepper anthracnose and apple bitter rot diseases in Korea

**DOI:** 10.3389/fmicb.2024.1429646

**Published:** 2024-11-28

**Authors:** DaYoung Kim, Jungyeon Kim, Younmi Lee, Kotnala Balaraju, Ye-Ji Hwang, Mi-Hwa Lee, Wonsu Cheon, Hye Yeon Mun, Chang Soo Lee, Yongho Jeon

**Affiliations:** ^1^Department of Plant Medicals, Andong National University, Andong, Republic of Korea; ^2^Agricultural Science and Technology Research Institute, Andong National University, Andong, Republic of Korea; ^3^Using Technology Development Department, Sangju, Republic of Korea; ^4^Diversity Conservation Research Department, Sangju, Republic of Korea; ^5^Biological Resources Research Department, Nakdonggang National Institute of Biological Resources, Sangju, Republic of Korea

**Keywords:** apple, biocontrol agent, *Colletotrichum* disease, pepper, *Streptomyces sporoverrucosus* B-1662, whole-genome sequencing

## Abstract

Fungi are the prominent phytopathogens that have significant impact on the productivity of agriculture worldwide. *Streptomyces* species have been extensively studied for the production of various bioactive metabolites. These metabolites have been used as biocontrol agents for the management of diseases caused by phytopathogenic fungi. The purpose of this investigation is to assess the efficacy of *Streptomyces sporoverrucosus* B-1662, an antagonistic agent in the control of red pepper anthracnose caused by *Colletotrichum acutatum* KACC 42403 and apple anthracnose caused by *Colletotrichum siamense* CGCP6 (GYUN-10348). On the basis of the morphological, and molecular characterization using 16S rRNA, the strain B-1662 was determined to be *S. sporoverrucosus*. The strain B-1662 exhibited antagonistic activity against seven fungal phytopathogens, including *C. acutatum* KACC 42403 and *C. siamense* CGCP6. The culture filtrates (CF) from B-1662 showed antifungal activity against all seven fungal pathogens with greater inhibition rate (%) in comparison with a control. The bacterial suspensions of B-1662 showed an excellent biological control effect on the red pepper anthracnose and apple bitter rot using an *in planta* assay. The anthracnose disease rate (%) was controlled by over 90% with B-1662 cell suspensions at 10^5^ to 10^7^ CFU/mL. Compared to a control, the strain B-1662 played a more effective role in controlling the anthracnose disease in field conditions in both years 2022 and 2023. From the effective solvent fractions, the effect compound (dibutoxybutane) has been isolated exhibiting with antifungal effect. The genetic base underlying the biocontrol traits of B-1662 was characterized using the whole-genome sequence of B-1662, which was compared with closely related strains. Consequently, these results collectively suggest that *S. sporoverrucosus* B-1662 can aid in the management of red-pepper anthracnose.

## Introduction

Red pepper (*Capsicum annum* L.) is one of the most important vegetables in Korea; however its yield and quality have been frequently limited by anthracnose disease, caused by the pathogen *Colletotrichum gloeosporioides* and *acutatum* (Kim et al., 1999; Park et al., 2006). Red pepper anthracnose can lead to significant reductions in crop yield and quality. Infected fruits may become disfigured, develop sunken lesions, and rot, rendering them unmarketable (Suprapta, 2022). This can result in financial losses for farmers and the agricultural industry as a whole. *Colletotrichum* species are responsible for their ability to infect a wide range of host plants, including red peppers (Liu et al., 2020) and apples (Kim C. H. et al., 2020), causing anthracnose. Anthracnose caused by several *Colletotrichum* spp. is considered as one of the most destructive diseases that reduce the production and quality of red pepper (Sang et al., 2011). Anthracnose of pepper has been found to be associated with mainly up to four *Colletotrichum* species: *C. truncatum* (Damm et al., 2009), *C. gloeosporioides*, *C. acutatum* and *C. coccodes* (Harp et al., 2008). Different *Colletotrichum* species are able to infect pepper fruits at different stages of maturity (Than et al., 2008). Taxonomically, *C. gloeosporioides* and *C. acutatum* have been described as species complexes (Cannon et al., 2012; Damm et al., 2009).

*Colletotrichum* species complex have also been reported to cause anthracnose disease in various fruits, including apples in Korea (Lee et al., 2007; Oo et al., 2018). In particular, it is associated with apple anthracnose (Kim et al., 2018), with *Colletotrichum fructicola* and *C. siamense* reported to be the causal agents of apple anthracnose in South Korea (Kim et al., 2018; Park et al., 2018). Apple is one of the major fruit crops commercially cultivated in South Korea (Korea Rural Economic Institute [KREI], 2018). Anthracnose has been reported as one of the major diseases of the apple in the South Korea, causes major losses to the farmers in reducing the fruit quality and which implies low market quality (Cheon and Jeon, 2015; Kim et al., 2018). Anthracnose is also known as bitter rot, and is characterized by sunken lesions on apples and is responsible for their decay and reduction in quality. It is not possible to sell fruit affected by this disease on the market; thus, apple growers face severe economic losses if they cannot control it.

Development of resistant cultivars is expected to be effective in controlling the disease; however the resistance against *Colletotrichum* spp. is often broken down under field conditions (Park, 2007). Therefore, synthetic fungicides are generally used by the most of the farmers to control anthracnose disease; however, the continuous application of chemical pesticides causes health hazardous to the environment. Therefore, other alternative measures are needed to overcome the problem of anthracnose disease in red peppers and apples. Owing to the increased consumers for organic agricultural products in Korea, organic farming systems are becoming the most popular among the farmers. To develop organic farming, it is important to provide bio-control agents (BCA)s to manage plant diseases. The use of BCA to control plant fungal diseases may reduce the use of synthetic chemical fungicides. Several BCA have been reported to possess inhibitory activities against plant pathogens.

Considering the growing desire of consumers for healthy foods, it is necessary to isolate and identify high efficiency and safety BCAs to control diseases. Among these BCAs, *Streptomyces* are considered as potential agents against fungal diseases because of high efficiency of producing functional metabolites (Getha and Vikineswary, 2002; Danial et al., 2020). Recently, *Streptomyces* species are drawing intensive attentions in controlling the anthracnose of strawberry (Li et al., 2021). Some bioactive metabolites such as desertomycin, spectinomycin, nigericin and validamycin isolated from *Streptomyces* spp. exhibited the strong antimicrobial potential (Jogaiah et al., 2016; Kim J. D. et al., 2020). Similarly, some other studies have showed that *Streptomyces* sp. JBS5-6 and *S. lunalinharesii* B-03 had strong inhibition abilities for spore germination and hyphal development of *Fusarium oxysporum* TR4 (Jing et al., 2020). The biocontrol mechanism of *Streptomyces* on plant diseases includes antagonism, competition, and inducing plant resistance, and it is one of the main sources of known bioactive substances (Vurukonda et al., 2018). Several species of actinobacteria were reported to have strong antagonistic activity against various species of *Colletotrichum* infecting a variety of crops. Thilagam and Hemalatha (2019) reported that *Streptomyces violaceoruber* reduced the incidence of chili anthracnose by inhibiting the spore germination and mycelial growth of *Colletotrichum capsici*. *Streptomyces ambofacines* S2 extract completely inhibited the expression of anthracnose symptoms of *Colletotrichum gloeosporioides* in red peppers (Heng et al., 2015). Actinobacteria not only prevent post-harvest pathogenic infection but also prolong the shelf life of a variety of crops without upsetting the natural balance (Renuka et al., 2023).

The objectives of the present study were (i) to screen antagonisitic activity against fungal phytopathogens, (ii) to test the isolated *S. sporoverrucosus* B-1662 and its culture filtrate (CF) against fungal pathogens, (iii) to test the biocontrol efficacy of the B-1662 against red pepper anthracnose and bitter rot of apple *in vivo*, (iv) to assess the biocontrol efficacy of the B-1662 against red pepper anthracnose in field conditions, (v) to extract the secondary metabolites and identification of bioactive compound from B-1662.

## Materials and methods

### Antagonistic bacterium and fungal pathogens

*Streptomyces sporoverrucosus* B-1662 was selected as a representative strain among the microorganisms isolated by Freshwater Bioresources Culture Collection (FBCC) from a soil sample collected at 169-2, Yeonsu-ri, Yongmun-myeon, Yangpyeong-gun, Gyeonggi-do (37°31′03.0″N, 127°38′28.7″E), for its confirmed antagonistic effects against several plant pathogenic microorganisms. The isolate B-1662 was maintained on tryptic soy agar (TSA) plates at 4°C in the refrigerator. For long-term storage, spore suspensions were stored in glycerol 20% (v/v) at −80°C in an ultralow freezer (Thermo fisher scietific, US). For preparing bacterial suspensions, culture from −80°C was grown on TSA plates at 28°C for 24 h, and single colonies were transferred to 50-mL falcon tube containing 30 mL tryptic soy broth (TSB) and incubated at 28°C for 24 h under shaking conditions (180 rpm). Various phytopathogenic fungi used in our study are as follows: *Colletotrichum acutatum* (KACC 42403), *C. coccodes* (KACC 48737), *Fusarium oxysporum f.sp lycopersici* (KACC 40043) were obtained from Korean Agricultural Culture Collection (KACC). *Colletotrichum siamense* CGCP6 (GYUN-10348), *Alternaria alternata* MGFJ029 (GYUN-10958), *Botryosphaeria dothidea* USFJ2 (GYUN-11058), and *Colletotrichum fructicola* ANBA7 (GYUN-10195), were isolated from our laboratory. All fungal isolates were cultured on potato dextrose agar (PDA, Difco, USA) plates at 25°C for 7 days and then stored at 4°C.

### Cultural, morphological characteristics and molecular identification of *S. sporoverrucosus* B-1662

For cultural characterization, the isolate B-1662 was inoculated on different ISP (International Streptomyces Project) media (ISP-1, ISP-2, ISP-3, ISP-4, and ISP-5) for 3 days incubation at 28°C. The cells were characterized based on color of the bacterial colony and soluble pigment production in the medium. The morphological characteristics were assessed by scanning electron microscope (SEM) of 3 day old cultures grown on ISP-3 medium. The strain B-1662 cultured for 3 days at 28°C on ISP-3 medium was fixed in 2% glutaraldehyde in 0.1 M phosphate butter (pH 7.2) for 3 hours at 4°C. Subsequently, the samples were rinsed three times, each for 10 min with 0.1 M phosphate buffer. Then the samples were fixed with 1% osmium tetraoxide (OsO_4_) in 0.1 M sodium cacodylate buffer (pH 7.2) under cold (4*^o^*C) conditions for 1 hour. After fixation, the specimens were rinsed again in 0.1 M phosphate buffer with 3-10 min rinses at 10-min intervals. Then, the specimens were washed with 0.2 M sodium cacodylate buffer (pH 6.2) and dehydrated with an increasing concentration of ethanol washes from 50 to 100% at 10 min intervals (50%, 70%, 80%, 90% and 100%). Later the specimens were mounted on aluminum stubs using conductive double-sided carbon tape. The stubs were then lyophilized, and sputter coated with gold (5 nm thickness). Finally, the morphological features of the bacterial strain were observed using a scanning electron microscope (SEM, VEGA II LMU, Tokyo, and Japan).

The 16S rRNA gene of the selective isolate B-1662 was analyzed for molecular identification. The genomic DNA (gDNA) of B-1662 was extracted from the cells grown in R2A medium for 72 h at 28°C using a bacterial genomic DNA extraction kit (Takara, Seoul, Korea) as per the manufacturer’s instructions. The 16S rRNA gene was amplified using a polymerase chain reaction (PCR) with Taq DNA polymerase, and primers 27F (5′-AGA GTT TGA TCM TGG CTC AG-3′) and 1492R (5′-GGC TAC CTT GTT ACG ACT T-3′) were used for amplification.

The PCR reaction volume was 50 μL, containing 25 μL Taq DNA polymerase and reaction buffer mix, 1 μL forward primer (10 μM), 1 μL reverse primer (10 μM), 1 μL genomic DNA template, and 22 μL nuclease-free water. The thermal cycling conditions were as follows: denaturation at 95°C for 5 min, followed by 30 cycles at 95°C for 30 s, annealing at 55°C for 30 s, and extension at 72°C for 90 s. The reaction mixture was held at 72°C for 5 min and then cooled to 4°C at the end of the cycle.

The PCR product was purified using a PCR gel purification kit (BIOFACT Co., Seoul, South Korea), according to the manufacturer’s instructions. The purified PCR product was sequenced using an automated sequencer (Genetic Analyzer 3130; Applied Biosystems, Carlsbad, CA, United States) with the same primers. The sequence was compared with reference bacterial species contained in a genomic database using the Basic Local Alignment Search Tool (BLAST) from the National Center for Biotechnology Information (NCBI). Sequence alignment and phylogenetic tree construction were accomplished using the MEGA-X software (Biodesign Institute, Tempe, AZ, United States) and the neighbor-joining method.

### *In vitro* antagonistic activity of *S. sporoverrucosus* B-1662 against phytopathogenic fungi

*S. sporoverrucosus* B-1662 was tested for *in vitro* antagonistic activity against seven fungal phytopathogens, including *C. acutatum* KACC 42403, *C. coccodes* KACC 48737, *Fusarium oxysporum* f.sp. *lycopercisi* KACC 40043, *C. siamense* CGCP6, *Alternaria alternata* MGFJ029, *Botryosphaeria dothidea* USFJ2, and *C. fructicola* ANBA7 using a dual culture plate assay. Fungal pathogens were cultured onto PDA plates (90 mm diameter) at 25°C for 10 days. The bacteria B-1662 was cultured in R2A agar plates at 28°C for 3 days. A mycelial plug was taken from the fully grown PDA plate using a sterile Cork borer (5 mm in diameter) and inoculated onto PDA medium supplemented with peptone (PDK) on one side 1.5 cm away from the edge, while the bacterium was streaked on the opposite side. PDA plates inoculated with fungal disks alone were used as controls. The inhibition zones of fungal pathogens, such as *C. acutatum* KACC 42403, *C. coccodes* KACC 48737, *F. oxysporum* f.sp. *lycopercisi* KACC 40043, *C. siamense* CGCP6, *A. alternata* MGFJ029, *B. dothidea* USFJ2, and *C. fructicola* ANBA7 were measured 16, 10, 11, 11, 15, 5, and 11 days, respectively, after incubation at 25°C. The inhibition of mycelial growth rate (%) was calculated using the following formula:

Inhibition of mycelial growth (%) = [(1−mycelial growth of treatment/mycelial growth of control)] × 100.

### Antifungal activities of culture filtrate of strain B-1662

The *Streptomyces* strain B-1662 was cultured in 250 mL flasks containing 100 mL of TSB under shaking conditions (180 rpm) at 28°C for 6 days. During culturing, 2 mL of the culture broth was collected continuous 6 days. The culture broth was then centrifuged at 13,000 × *g* for 5 min at 4°C, and the resulting supernatant was sterilized through a syringe filter (pore size; 0.22 μm). This was used as a cell-free culture filtrate (CF). Mycelial plugs (5 mm in diameter) of seven pathogens, such as *C. acutatum* KACC 42403, *C. coccodes* KACC 48737, *F. oxysporum* KACC 40043, *C. siamense* CGCP6, *A. alternata* MGFJ029, *B. dothidea* USFJ2, and *C. fructicola* ANBA7 were placed on the center of PDK plates. The sterile paper disks (6 mm diameter) impregnated with 20 μL of the CF, were placed onto the same plate at four different sides. Paper disks impregnated with TSB were used as non-treated controls. Growth was measured 3 d after incubation at 25°C. Each treatment consisted of three replicates and the experiment was performed at least twice. The inhibition of mycelial growth rate (%) was calculated using the following formula:

Inhibition of mycelial growth (%) = [(1−mycelial growth diameter of treatment/ mycelial growth diameter of control)] × 100.

### Effect of B-1662 cell suspensions on conidial germination of *C. acutatum*

To determine the potential role of B-1662 in inhibiting the conidia germination of *C. acutatum in vitro*, conidia were harvested from PDA plates after 10 days of cultivation at 25°C. The concentration of conidia was adjusted to 10^5^ spores/mL using a hemocytometer. The bacterial isolate B-1662 was cultured for 72 h at 28°C in TSB broth, and culture suspensions were prepared in three different concentrations (10^5^, 10^6^, and 10^7^ CFU/mL). The conidia germination and appressorium formations from *C. acutatum* ANUP-HJ were evaluated using a previously described (Kwak et al., 2012) on a slide glass surface treated with B-1662 bacterial suspensions. Briefly, the conidia suspensions (10 μL) were combined with bacteria suspensions (10 μL) at varying concentrations, and the resulting mixture was transferred to glass slides. Conidia suspensions without treating bacterial suspensions were considered as a control. We evaluated the germination of conidia and the development of appressorium in the B-1662 treatment during incubation at 25°C and different time duration (0, 12, 24, 32, and 48 h) in Petri dishes containing moist paper. The glass slides were observed under a light microscope (Olympus BX43, Olympus, Tokyo, and Japan). The experiment was performed three times in triplicates. The conidia germination rate (%) and appressorium formation rate (%) were calculated using the following formula:

Conidia germination rate (%) = (germination of treated conidia/germination of control) × 100.

Appressorium formation rate (%) = (count of treated appressorium/appressorium of control) × 100.

### Thermo-stability of antifungal compounds in CF of *Streptomyces sporoverrucosus* B-1662

To check the thermo-stability of the antifungal metabolites produced by *Streptomyces* sp. B-1662, CF was kept at different temperatures (20°C, 37°C, 50°C, 70°C, and 100°C) in water bath for one hour (Kaur et al., 2019). Photo stability was tested by exposing the CFs separately to ultra violet (UV) light in a clean bench for 1 hour. All the treated samples were then checked for the residual activity of against *C. siamense* GYUN-10348.

### Extraction of secondary metabolites and their antifungal activity *in vitro*

The B-1662 strain was inoculated into 2000 mL Erlenmeyer flasks containing 1000 mL of TSB. After incubation at 28°C for 3 days under shaking conditions at 180 rpm, the culture broth was centrifuged at 8,000 × *g* for 20 min and the supernatant was collected. The supernatant was sequentially mixed with different organic solvents, such as hexane, ethyl acetate, chloroform and n-butanol in 1:3 ratio (supernatant: solvent) for 24 h. Later, organic solvent was separated using a separating funnel and concentrated under reduced pressure using a rotary vacuum evaporator. The antifungal activity of the active fraction extracted from the culture supernatant of strain B-1662 was assessed using the following procedure: initially, 300 μL of the concentrated solvent fraction was added to 300 mL of PDA medium before solidification, thoroughly mixed, and then poured into sterilized Petri dishes to solidify. Then, a fully grown fungal pathogenic mycelial plug (5 mm diameter) of *C. acutatum* CGCP6 was placed on the center of the Petri dish and incubated at 25°C for 9 days. The diameter of the mycelium (mm) was then measured to determine the antifungal activity. The inhibition of mycelial growth rate (%) was calculated using the following formula:

Inhibition of mycelial growth (%) = [(1−mycelial growth of treatment/mycelial growth of control)] × 100.

### Identification of bioactive compounds from *Streptomyces sporoverrucosus* B-1662

The chemical compounds in *Streptomyces* extracts were identified using a gas chromatography-mass spectrometry. Helium was injected at 1 mL/min as a carrier gas. The column temperature was set to 50°C for 3 min, then increased to 100°C at 5°C/min for 5 min, then to 250°C at 10°C/min for 35 min, and finally to 280°C at 10°C/min for 25 min. The mass spectrometer was run in electron ionization (EI) mode at 70 eV, with an interface temperature of 280°C, an ion source temperature of 240°C, a mass spectrometer acquisition delay time of 3.5 min, and a continuous scan from 33 to 550 m/z. Peaks were identified by comparing the mass spectra data to the spectral library of the National Institute of Standards and Technology (NIST) (Wang et al., 2013).

### Effect of dibutoxybutane at different concentrations on mycelial growth of *C. siamense*

The antifungal activity of dibutoxybutane at different concentrations was evaluated using the following procedure: dibutoxybutane at various concentrations from 6 μL (0.1 ppm) to 1,800 μl (30 ppm) was added to 60 ml of PDA medium before the solidification of the PDA medium, and mixed thoroughly, and then poured into sterilized petri dishes to solidify. Then, a fully grown fungal pathogenic mycelial plug (5 mm diameter) of *C. siamense* CGCP6 was placed on the center of the Petri dish and incubated at 25°C for 4 days. PDA plates without treating with dibutoxybutane were considered as control group. The diameter of the mycelium (mm) was then measured to determine the antifungal activity. The inhibition of mycelial growth rate (%) was calculated using the following formula:

Inhibition of mycelial growth (%) = [(1−mycelial growth of treatment/mycelial growth of control)] × 100.

### Cluster of orthologous genes (COG) function annotation and secondary metabolites of *S. sporoverrucosus* B-1662

B-1662 DNA was extracted using the DNeasy Ultra Clean Microbial kit (Qiagen, Hilden, Germany) according to the manufacturer’s instructions, and the whole genome sequencing was performed by Macrogen. Then, the genome of B-1662 was constructed *de novo* using Macrogene sequencing data, Macrogene sequencing data were assembled with Macrogene SMRT Analysis 2.3.0, using the HGAP2 protocol (Pacific Biosciences, United States). Clusters of orthologouse genes (COGs) functional annotation pipeline of whole genome assembles used in EzBioCloud genome database. Predicted protein-coding sequences (CDSs) were predicted by Prodigal 2.6.2 (Hyatt et al., 2010). Genes coding for tRNA were searched using tRNAscan-SE 1.3.1 (Schattner et al., 2005). The rRNA and other non-coding RNAs were searched by a covariance model search with Rfam 12.0 database (Nawrocki and Eddy, 2013). The CDSs were classified into groups based on their roles, with reference to orthologous groups (EggNOG 4.5)^1^ (Powell et al., 2014). This analysis of antibiotics and secondary metabolites was conducted with antiSMASH (version 7.0.0) (Weber et al., 2015). In addition, the BAGEL 4 online web server and BAGEL4 were used to detect potential bacteriocins in all the investigated genomes (van Heel et al., 2018).

### Biological control efficacy of B-1662 against apple bitter rot *in vivo*

To determine the effect of B-1662 cell suspensions and culture filtrate (CF) obtained from the bacterial culture of B-1662 against apple bitter rot, apple fruits (cv. Fuji) of similar size were surface-sterilized with 70% ethanol for 3 minutes, followed by 1% NaOCl for 3 min, rinsed three times with sterile distilled water (SDW), and air-dried in a laminar airflow chamber. Thus, surface-sterilized apple fruits were wounded by piercing them 1 to 2 mm deep with a sterile needle at nine sites. These wounded apples were treated with 20 μL of B-1662 cell suspensions (10^7^ CFU/mL), or CF obtained from 5 d after incubation and allowed to dry for 12 h. Later, the apples were inoculated with 20 μL of spore suspensions (5.3 × 10^5^ conidia/mL) of *C. siamense* CGCP6 at wounding sites. The fruits were kept in a plastic tray containing a wet towel at the bottom in order to maintain the humidity level at 25°C. Apples treated with only B-1662 but without inoculation of *C. siamense* CGCP6 spore suspensions were served as a control group. All treatments were performed in triplicate. The disease rate (%) and control efficacy were determined by measuring the disease index 7 d after inoculation. The disease index scale was established from 0 to 4, where 0 = no symptoms, 1 = 0-25% with disease lesions ≥ 2 mm; 2 = 25-50% with tissue showing disease lesions ≥ 4 mm; 3 = 50-75% with disease lesions < 8 mm; 4 = 75-100% with sunken lesions and spore production. The disease severity are calculated using the following formulae:

Disease severity (%) = Σ (Disease index × Number of fruits in that Disease index)/(Total Number of Fruits × Maximum Disease index) × 100.

### Effect of B-1662 treatment on *in vivo* suppression of anthracnose disease in red peppers

To investigate the effect of B-1662 on anthracnose disease suppression in red peppers, fresh and healthy red pepper fruits of similar sizes were surface-sterilized in 1% NaOCl for 1 min and rinsed twice with SDW (Sterile Distilled Water). This experiment was conducted according to the method developed by Kim et al. (2023). The fungal pathogen *C. acutatum* ANUP-HJ causes anthracnose in red peppers and was isolated from the disease infected red pepper fruits, cultured onto PDA plates at 25°C for 9 days, and used in our study for inoculation. Five wounds were made on each red pepper fruit using a sterile needle and B-1662 suspensions (10 μL) at three different concentrations (10^5^, 10^6^ and 10^7^ CFU/mL) were dropped on each wound, allowed to dry, and the fruits were inoculated with 10 μL of fungal pathogen spore suspension (1 × 10^5^ spores/mL) on each wound just by dropping. Red pepper fruits treated with TSB served as a control. All fruits were placed on square plates (40 cm × 40 cm) containing moist paper. The disease rate (%) was recorded based on disease rating after incubating the plates at 25°C for 9 days. The disease index scale was established from 0 to 4, where 0 = no symptoms, 1 = 0–25% with disease lesions ≥ 2 mm; 2 = 26–50% with tissue showing disease lesions ≥ 4 mm; 3 = 51–75% with disease lesions < 8 mm; 4 = 76–100% with sunken lesions and spore production. The disease severity(%) are calculated using the following formulae:

Disease severity (%) = (disease index × number of fruits with disease index)/(maximum disease index × total number of fruits) × 100.

### Biological control efficacy of B-1662 against red-pepper anthracnose in field conditions

To assess the effect of antagonistic B-1662 bacteria against red pepper anthracnose disease under field conditions, two-month-old healthy red pepper (cv. color king) seedlings were purchased from a local seedling market and transplanted into the field at a spacing of 40 × 100 cm. The experiment was conducted in a field at Andong National University, Korea for two years. The treatments were grouped as follows for the first year: control, chemical control, pyraclostrobin, foliar spray (FS) with B-1662 (FS B-1662), and foliar spray + soil drench with B-1662 (FS+SD B-1662). The control group was divided into three categories. The ‘Control’ group served as a baseline for directly comparing treatment effects without any intervention. The ‘Chemical control’ group was established to compare the efficacy of the treatments with the conventional fungicides commonly used by farmers. Lastly, ‘Pyraclostrobin,’ a registered reference fungicide in Korea, was included as a treatment group for comparison with the microbial treatments. Detailed application schedules and the information of the chemical fungicides are provided in Supplementary Tables 1–3. Chemical control and Pyraclostrobin groups were using a foliar spray method. For the foliar spray treatment group, 250 mL of bacterial suspension (10^6^ CFU/mL) per plant was used, whereas 500 mL/plant was used for the soil drench treatment group. After transplantation in May, red pepper fruits developed in July, and then all treatments were administered 6 times at 10-day intervals (Supplementary Table 1). The first treatment was applied 49 days after transplantation (June 24, 2022). The disease rate (%) was calculated based on disease infected red pepper fruits harvested at two times corresponding to 98 and 112 days after transplantation (corresponding respectively to August 12, and August 26, 2022).

In the second year, the treatments were grouped as follows: control, chemical control, pyraclostrobin, B-1662, cross spraying of chemical and B-1662 (CS), and mixed spraying of chemical + B-1662 (MS). B-1662 bacterial cell suspensions prepared at a concentration of 10^6 CFU/mL were used for all treatments. Pyraclostrobin was diluted according to the guidelines provided by the Korea Crop Protection Association, with a field-use concentration of 5 mL per 20 L of water. For the mixed treatment groups (CS and MS), both solutions were diluted to maintain the same final field-use concentrations when combined. The field was irrigated every 10 days. The first treatment was applied 63 days after transplantation (July, 07, 2023), and a total of five treatments were administered at 12-day intervals until August, 21, 2023 using a foliar spray method (the detailed treatment schedule is provided in Supplementary Table 2). The disease rate (%) was calculated based on disease infected red pepper fruits harvested at three different times corresponding to 87, 101 and 118 days after transplantation (corresponding respectively to July 31, August 14, and August 31, 2023). The final harvest of fruits for evaluating the BCA effect was performed 10 days after the last treatment, on August 31, 2023. The disease severity (%) was determined using the following formula:

Disease severity (%) = (Number of diseased fruits/total number of fruits) × 100.

### Statistical analysis

Analysis of variance (ANOVA) was performed on R studio (R Core Team, 2023). Significant differences between treatment means were determined using the least significant difference (LSD) at *p* < 0.05. All the experiments were performed at least twice.

## Results

### Morphological characteristics and identification of *Streptomyces* sp. B-1662

**FIGURE 1:**
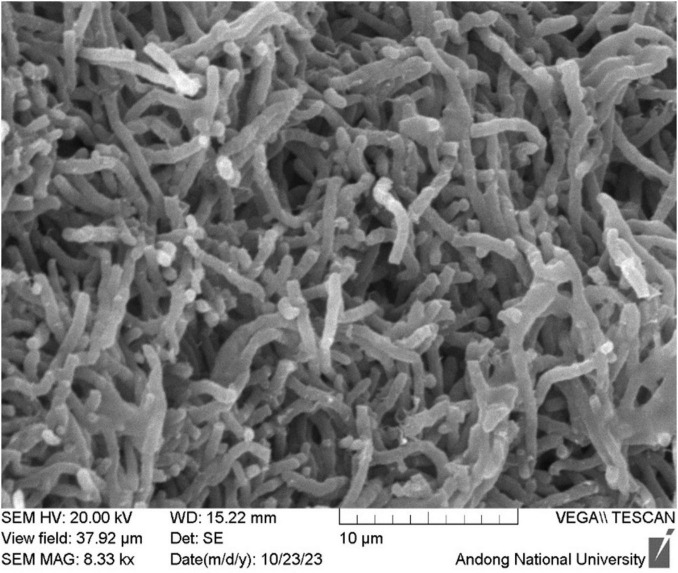
Scanning electron micrographs of the spore morphology of *Streptomyces* sp. B-1662 on IPS 3 medium after incubation at 28°C for 3 days, which showed the aerial hyphae, non-fragmented, and most spores were rod-shaped with a fragmented spore chain and smooth surface.

When the isolate B-1662 was cultured on different ISP media (ISP-1, ISP-2, ISP-3, ISP-4, ISP-5) for 3 days at 28°C, it exhibits white to yellow soluble pigments on ISP-1 and ISP-2 media; while red-brownish white and soluble pigments on ISP-3 medium (Supplementary Figure 1). The cultural characteristics of strain B-1662 were summarized in Supplementary Table 4. The strain B-1662 presented the typical morphology of a Gram-positive, lamentous. Scanning electron microscopy (SEM) revealed that the aerial hyphae were non-fragmented and most spores were rod-shaped and smooth surface (Figure 1). Based on cultural and morphological characteristics, the strain B-1662 belongs to the genus *Streptomyces* according to Nonomura (1974) key (1974).

**FIGURE 2:**
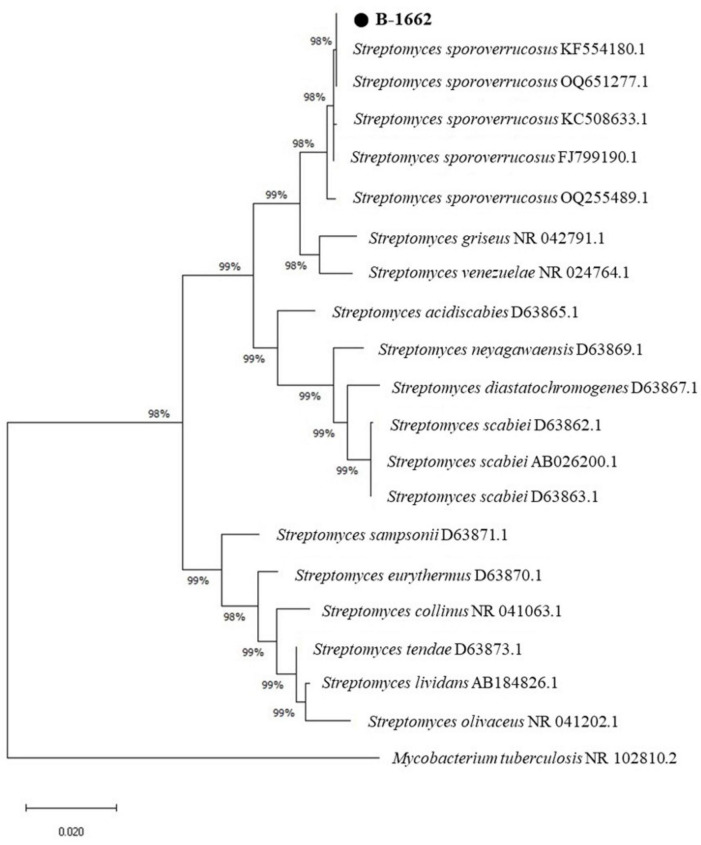
Neighbor-joining phylogenetic tree based on 16s rRNA gene sequence comparing *Streptomyces* B-1662 strain with other *Streptomyces* species collected from NCBI-BLAST. The numbers on the branches indicate the percentage bootstrap values of 1000 replicates. The Neighbor-joining phylogenetic tree was generated by the MEGA-X program. The scale bar indicates 0.02 Substitutions per nucleotide position.

The B-1662 was initially characterized using 16S rDNA gene sequencing, and the sequence obtained was submitted to GenBank under the accession number (OR781529). The BLAST analysis indicated that the isolate B-1662 belonged to the genus *Streptomyces*. In the phylogenetic tree, the B-1662 isolate was grouped with other *Streptomyces* spp. was closely related to *S. sporoverrucosus*, with 99.79% similarity. Phylogenic analysis based on 16S rRNA gene sequences using the neighbor-joining methods was shown in Figure 2); hence, molecular traits confirmed this species as *S. sporoverrucosus*. The isolate was found in a phylogenetic tree among other *Streptomyces* species closely related to *S. sporoverrucosus*.

### Antagonistic activity of the strain B-1662 against phytopathogenic fungi

**FIGURE 3:**
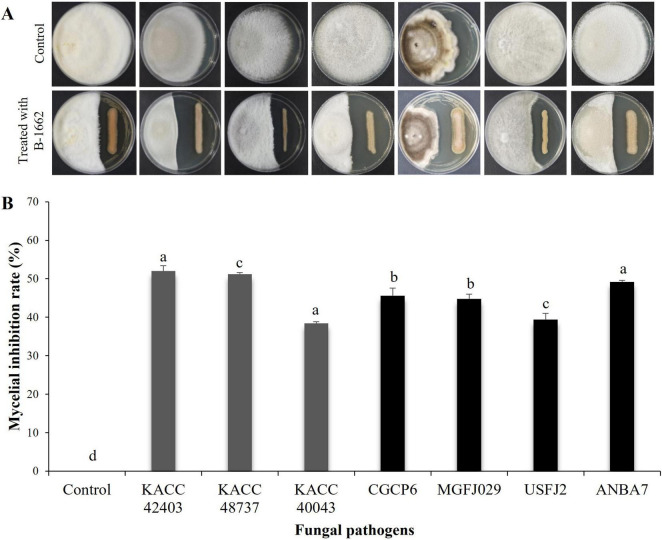
*In vitro* antagonistic activity of *Streptomyces* sp. B-1662 against fungal phytopathogens using a dual culture plate assay. The mycelial plug of a pathogenic fungus from a 5-day-old PDA plate was inoculated onto PDK medium, while B-1662 was streaked on the opposite side of the same plate. The plates inoculated with only mycelial discs alone served as the control group. **(A)** The inhibition zones of various fungal pathogens, such as *Colletotrichum acutatum* KACC 42403, *C. coccodes* KACC 48737, *Fusarium oxysporum* f.sp. *lycopercisi* KACC 40043, *C. siamense* CGCP6, *Alternaria alternata* MGFJ029, *Botryosphaeria dothidea* USFJ2, and *C. fructicola* ANBA7 were measured 16, 10, 11, 15, 15, 5, and 13 days, respectively, after incubation at 25°C; the left and right colonies on the plates are phytopathogenic fungus and B-1662, respectively. **(B)** The percentage of mycelial growth inhibition rate was calculated from the inhibition zone. The experiment was performed twice in triplicate. Bars with the same letters do not differ from each other according to the least significant difference (LSD) (*P* < 0.05).

The strain B-1662 showed significantly inhibitory effects on the mycelial growth of the tested phytopathogenic fungi *C. acutatum* KACC 42403, *C. coccodes* KACC 48737, *F. oxysporum f.sp. lycopercisi* KACC 40043, *C. siamense* CGCP6, *A. alternata* MGFJ029, *B. dothidea* USFJ2, and *C. fructicola* ANBA7 using a dual culture plate assay (Figure 3). B-1662 significantly suppressed mycelial growths of *C. acutatum* KACC 42403 and *C. coccodes* KACC 48737 following incubation at 25°C for 16 and 10 days, respectively, when compared to other pathogens. Compared to the control, the mycelial growths of *C. acutatum* KACC 42403, *C. coccodes* KACC 48737, *F. oxysporum f.sp. lycopercisi* KACC 40043, *C. siamense* CGCP6, *A. alternata* MGFJ029, *B. dothidea* USFJ2, and *C. fructicola* ANBA7 were inhibited by 52, 51.2, 38.4, 45.6, 44.8, 39.4, and 49.2%, respectively, implying a broad antifungal spectrum.

### Antifungal activity of culture filtrates of the strain B-1662

**FIGURE 4:**
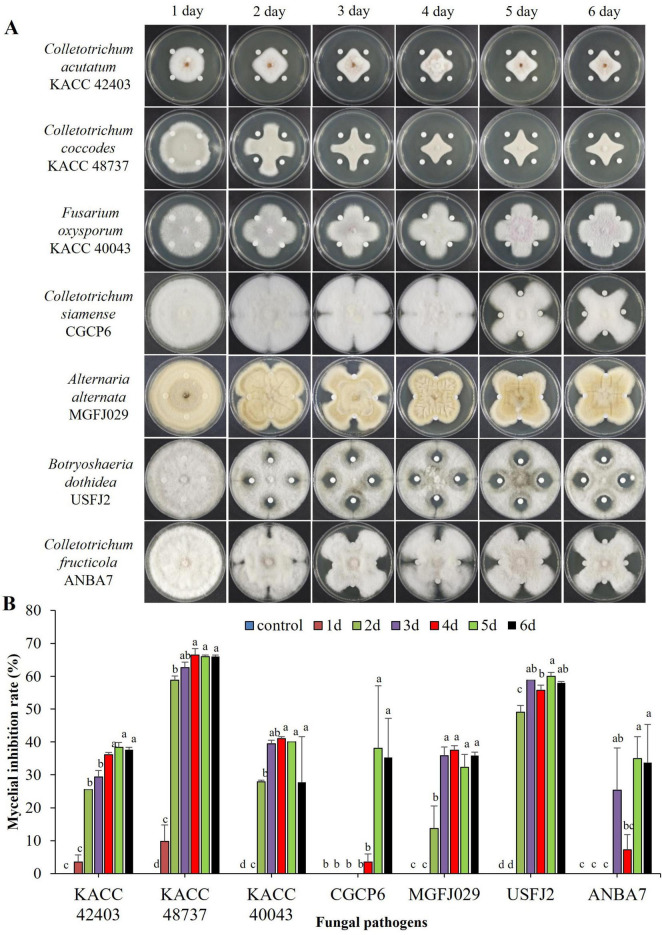
Inhibitory effect of culture filtrate (CF) of antagonistic *Streptomyces* sp. **(A)** B-1662 on growths of various phytopathogenic fungi under *in vitro* conditions. Paper disks impregnated with TSB were used as a non-treated control. The diameter of mycelial growths of fungal pathogens on PDK plates was recorded 12 d after incubation at 25°C. **(B)** The percentage of mycelial growth inhibition rate was calculated from the inhibition zone. The experiment was performed twice in triplicate. Bars with the same letters do not differ from each other according to the least significant difference (LSD) (*P* < 0.05).

The inhibitory effect of the culture filtrate (CF) of the strain B-1662 was evaluated against the growth of all seven fungal pathogens. The B-1662-CF-treated fungi exhibited a decrease in colony diameter in comparison to the untreated control (Figure 4). Treatment of CF from 5-day-old B-1662 resulted in a more pronounced inhibition of all pathogens. However, there was no significant difference between the inhibition rates of CF treatments administered after 5 and 6 days. The resulting inhibition rates of mycelial growths were 38.35%, 66.03%, 40%, 38.03%, 32.27%, 60%, and 34.93% for *C. acutatum* KACC 42403, *C. coccodes* KACC 48737, *F. oxysporum f.sp. lycopercisi* KACC 40043, *C. siamense* CGCP6, *A. alternata* MGFJ029, *B. dothidea* USFJ2, and *C. fructicola* ANBA7, respectively, in the B-1662-CF treatment. The mycelial growth of KACC 48737 was inhibited to a greater level with an inhibition rate of 66.03% compared to the inhibition rates of other pathogens, while the lower level of mycelial growth of *A. alternaria* (MGFJ029) was observed with an inhibition rate of 32.27%. The TSB, used as a negative control, exhibited no inhibitory effects on the mycelial growth of the tested pathogens. Despite commencing antibiotic production on the second day, the strain B-1662 did not exhibit its maximum inhibition rates until the fifth day. This finding suggested that the suppression of mycelial growth of fungal pathogens by the CF derived from the strain B-1662 proved that it secreted antifungal bioactive secondary metabolites which played a role in the reduction of mycelial growth of fungal pathogens.

### Effect of B-1662 culture suspensions on conidia germination of *C. acutatum* under *in vitro* conditions

**FIGURE 5:**
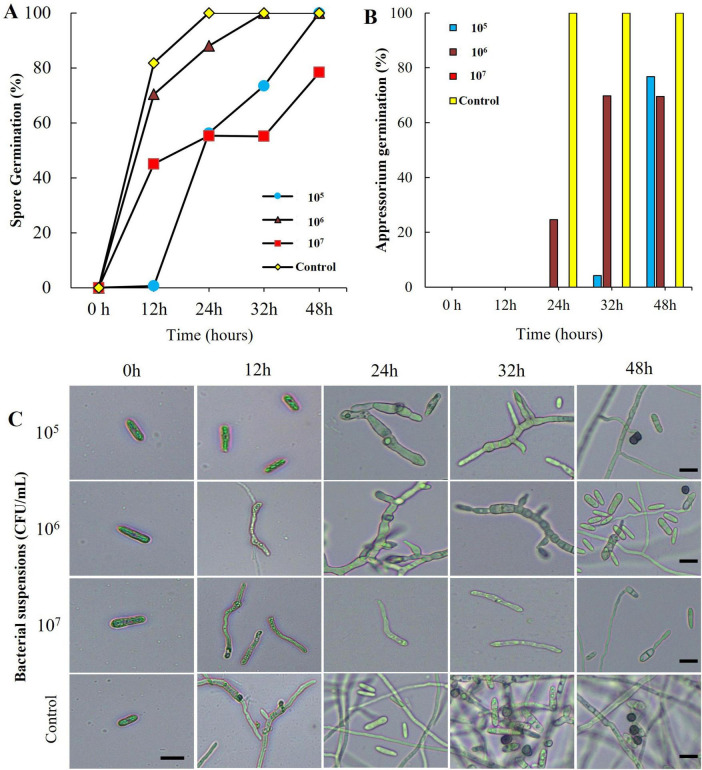
Effect of bacterial suspensions of *Streptomyces sporoverrucosus* B-1662 treatment on conidia germination rate (%) of *C. acutatum* and microscopic observation: **(A)** Conidial germination rate (%) was suppressed by bacterial suspensions, while the germination rate (%) was increased in the non-treated control; and **(B)** appressorium formation rate (%) at different time intervals were recorded. **(C)** Microscopic observations of *C. acutatum* spore germination after treatment with B-1662 cell suspensions at various concentrations (10^5^, 10^6^, and 10^7^ CFU/mL) during the incubation period from 0 to 48 h. The germination counting was carried out using a hemocytometer. The percentage at a given time was recorded by observing at least 200 conidia for each treatment. Bar = 10 μm. The experiment was performed two times in triplicate producing similar results.

When conidial spores of *C. acutatum* ANUP-HJ were treated with B-1662 culture suspensions at various concentrations *in vitro*, varying levels of damage in conidia germination were observed, which were compared to the non-treated control. Analysis of conidial spore germination revealed that conidia treated with B-1662 cell suspensions exhibited a higher percentage of inhibition of spores after 16 h of incubation in comparison to untreated conidia. The conidial germination rate in the B-1662 culture suspension at 10^5^, 10^6^ and 10^7^ CFU/ml was 44.39, 76.54 and 45.54%, respectively, after 16 h of incubation (Figure 5). In contrast, the spore germination rate in the untreated control group increased significantly. At 48 h, all conidia in the water-treated control had germinated and appressorium had formed via germ tubes; this did not occur with conidia treated with B-1662 at 10^7^ CFU/ml. This finding indicates that B-1662 cell suspensions effectively inhibited the germination of conidial germination of *C. acutatum* ANUP-HJ (Figure 5).

### Stability of antifungal compounds in culture filtrates of *S. sporoverrucosus* B-1662

The stability of antifungal compounds present in the culture supernatant of *Streptomyces* sp. B-1662 was evaluated under various physical stress conditions (Supplementary Table 6). To assess this stability, the antifungal activity of *Streptomyces* sp. B-1662 was determined based on the inhibition of the mycelial growth of *C. siamense* CGCP6. The antifungal compounds produced by B-1662 were found to be completely stable at temperatures up to 37°C for one hour, retaining over 30% activity even at 50°C and 70°C compared to the control group (Supplementary Figure 2). Moreover, the antifungal activity remained above 30% after one hour of heating the culture supernatant.

### Antifungal activity of solvent extracts from *S. sporoverrucosus* B-1662 against pathogenic fungi and identification of bioactive compound

**FIGURE 6:**
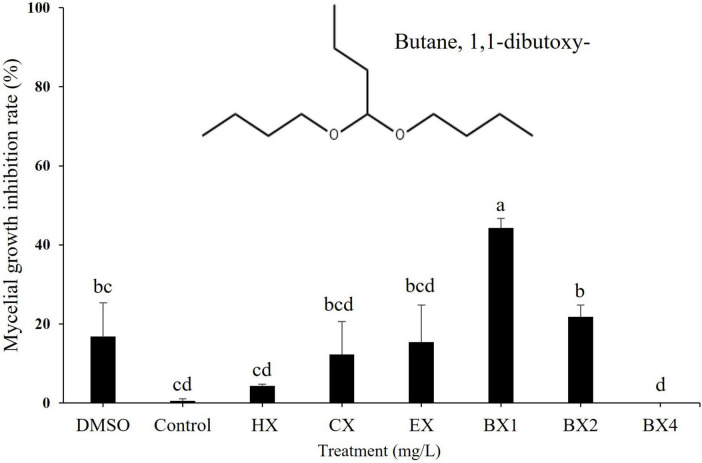
Effect of various solvent fractions of B-1662 against the mycelial growth of *Colletotrichum siamense* CGCP6 *in vitro*. Mycelial growth inhibition rate (%) of *Colletotrichum siamense* CGCP6 on PDAA medium was recorded 9 d after incubation at 25°C in comparison with a non-treated control. The experiment was performed two times in triplicates. Bars with the same letters do not differ from each other according to the least significant difference (LSD) (*P* < 0.05). HX, Hexane; CX, Chloroform; EX, Ethyl acetate; BX1, Butanol layer 1; BX2, Butanol layer 2; BX4, Butanol layer 4.

Among all the solvent extracts of *S. sporoverrucosus* B-1662 tested, n-butanol solvent fraction extract at different concentrations exhibited antifungal activity against the growth of *C. siamense* CGCP6 at a greater level than the other solvent extracts under *in vitro* conditions (Figure 6). The mycelial growth inhibition rate (%) was observed as 44.26% 9 days after incubation. However, chloroform and ethyl acetate extracts have exhibited the mycleial growth inhibition rates at low rates, with 12.2% and 15.4%, respectively. Based on these results, the sample was subjected for further investigation using a GC-MS analysis.

For the identification of bioactive compounds produced by *S. sporoverrucosus* B-1662, GC-MS analysis was conducted. The GC-MS analysis revealed the presence of a compound known as ‘Butane, 1,1-dibutoxy-’ which has been previously reported to be associated with antifungal activity (Figure 6). This compound was isolated from the first volatile fraction layer of the n-butanol layer, where high antifungal activity was observed. It was not detected in the other organic solvent layers (Supplementary Table 5). Based on these findings, it is presumed that the bioactive compounds responsible for the antifungal activity exhibited by *S. sporoverrucosus* B-1662 are well soluble in n-butanol.

### Effect of dibutoxybutane at different concentrations

*In vitro* tests were conducted to determine the efficacy of various concentrations of the commercially available compound ‘dibutoxybutane’ against the mycelial growth of pathogenic fungus *C. siamense*. The inhibition rate of fungal mycelial growth is proportional in magnitude to the dibutoxybutane concentration. The maximum concentration, which exhibited a 100% inhibition rate, was determined to be 30 μg/mL (Supplementary Figure 3). The resulting inhibition rates of mycelial growths were 0.85, 4.27, 7.69, 25.64, 71.79, and 100% for 0.1, 0.3, 1.0, 3.0, 10, and 30 μg/mL concentrations, respectively, in the dibutoxybutane treatment. In the absence of dibutoxybutane, plates exhibited no inhibition of mycelial growth. At concentrations exceeding 10 μg/mL, *C. siamense* growth was significantly inhibited. Dibutoxybutane exhibited IC_50_ value of 6.79 ± 2 μg/mL towards *C. siamense*. The PDA plate with 10 μg/mL of dibutoxybutane showed slight growth, while 30 μg/mL showed no growth. This result establishes that the butanol fraction of B-1662-derived compound dibutoxybutane inhibits the growth of *C. siamense*.

### Cluster of orthologous genes (COG) annotation, and secondary metabolites of B-1662

The strain B-1662 consists of four contigs. The whole-genome size is 8,254,451 bp, with a G+C content of 72.4% and 7,262 predicted protein-coding sequences (CDSs). All predicted CDSs of B-1662 were compared with the clusters of orthologous genes (COG) databases to identify homologous amino acid sequences. Each functionally annotated protein was assigned a COG number, representing a class of proteins; then, the proteins were subjected to functional clustering analysis according to the COG function. Totally, 172 and 103 genes were associated with secondary metabolites biosynthesis, transport, catabolism, and defense mechanisms, respectively (Supplementary Figure 4 and Supplementary Table 7). A total of 27 biosynthetic gene clusters (BGC) were identified, three of these clusters are associated with the production of non-ribosomal peptides (Supplementary Figure 4B). Five secondary metabolites with 100% similarity were identified using anti-SMASH. Contig 1 is 7,458,343 bp and has 6,474 CDSs, with 27 tRNAs and 21 rRNAs contig 2 is 532,210 bp with 513 CDS and 3 tRNAs, contig 3 is 157,211 bp with 148 CDS, and contig 4 is 107,687 bp with 127 CDS (Supplementary Figure 5). A total of 7,262 CDS, 90 tRNA, and 21 rRNA genes were found in the four contigs (Supplementary Table 7). To identify highly promising genes associated with the biosynthesis of secondary metabolites in bacterial genomes, anti-SMASH version 7.0.0 was employed for the rapid genome-wide identification, annotation, and analysis of BGCs involved in secondary metabolite biosynthesis in B-1662.^2^

### Biological control efficacy of B-1662 against red pepper anthracnose and apple bitter rot

**FIGURE 7:**
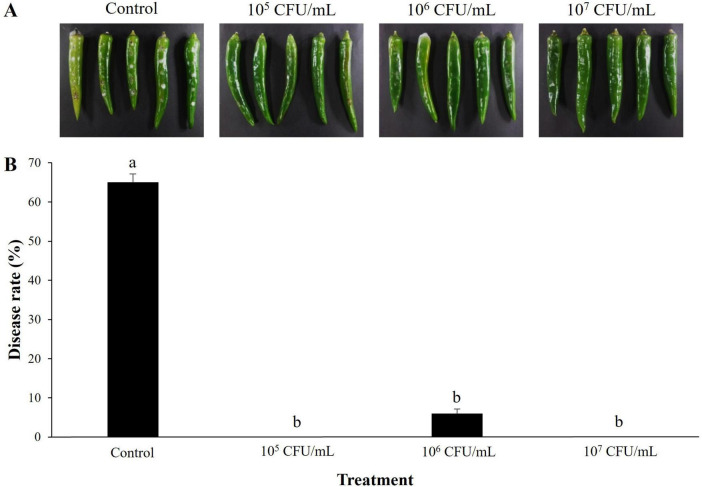
Effect of *S. sporoverrucosus* B-1662 cell suspensions on suppression of disease rate (%) of anthracnose caused by *C. acutatum* on red-pepper fruits *in vivo*. **(A)** Wounded red-pepper fruits were treated with B-1662 culture suspensions at three different concentrations (10^5^, 10^6^, and 10^7^ CFU/mL), followed by inoculation with *C. acutatum* ANUP-HJ conidia suspensions (5 × 10^5^ conidia/mL). Red-pepper fruits treated with TSB served as a control. **(B)** The results were compared with a non-treated control 9 days after incubation at 25°C. The experiment was performed two times with three replicates. Bars with the same letters do not differ from each other according to the least significant difference (LSD) (*P* < 0.05).

The preventive effect of *S. sporoverrucosus* B-1662 against the anthracnose pathogen *C. acutatum* ANUP-HJ was evaluated. Disease severity (%) was reduced to a greater level at all the three concentrations of B-1662 suspensions when compared to the non-treated control group 7 days after incubation (Figure 7). The disease severity was dramatically reduced to 0% in B-1662-treated plants at 10^5^ and 10^7^ CFU/mL, while the disease severity has been recorded as 6% at 10^6^ CFU/mL, whereas it has been observed as 65% in a non-treated control.

**FIGURE 8:**
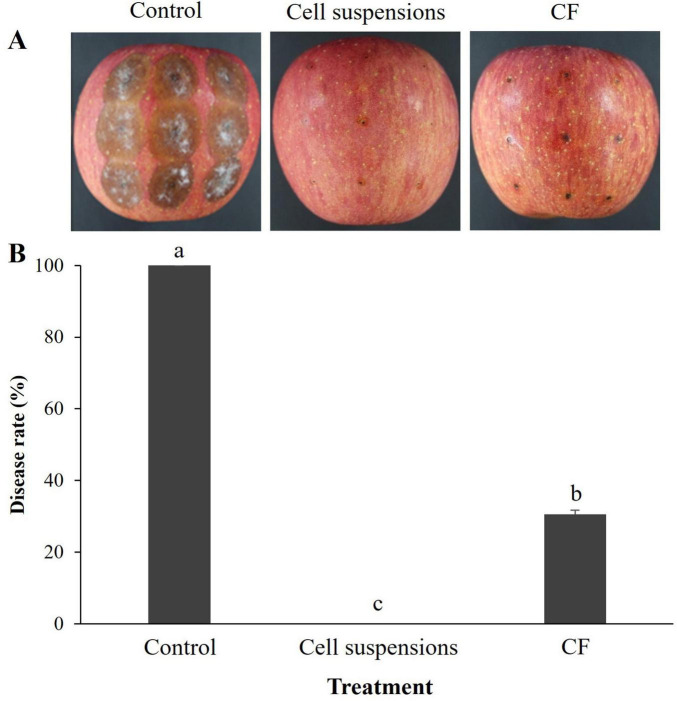
Effect of *S. sporoverrucosus* B-1662 cell suspensions and its culture filtrate (CF) on control of apple bitter rot caused by *C. siamense* in wounded apples. **(A)** B-1662 cell suspensions (10^6^ CFU/mL) and CFs (obtained from 5 days after incubation) were dropped on the detached apple fruits, followed by inoculation with *C. siamense* conidia suspensions (5.3 × 10^5^ conidia/mL) after 1 day. **(B)** The results were compared with a non-treated control 11 days after incubation at 25°C. The experiment was performed two times with three replicates. Bars with the same letters do not differ from each other according to the least significant difference (LSD; *p* < 0.05).

In the case of preventive effect of B-1662 on control of apple bitter rot caused by *C. siamense*, B-1662 cell suspensions and culture filtrate (CF) significantly (*p* < 0.05) reduced disease severity (%) when compared to the non-treated control (Figure 8). Apples treated with B-1662 cell suspensions exhibited the greatest disease suppression without indication of disease severity. The disease severity in apples treated with CF was 30.55%, compared to 100% in control apples.

### Effect of B-1662 on disease control of red pepper anthracnose in field conditions

**FIGURE 9:**
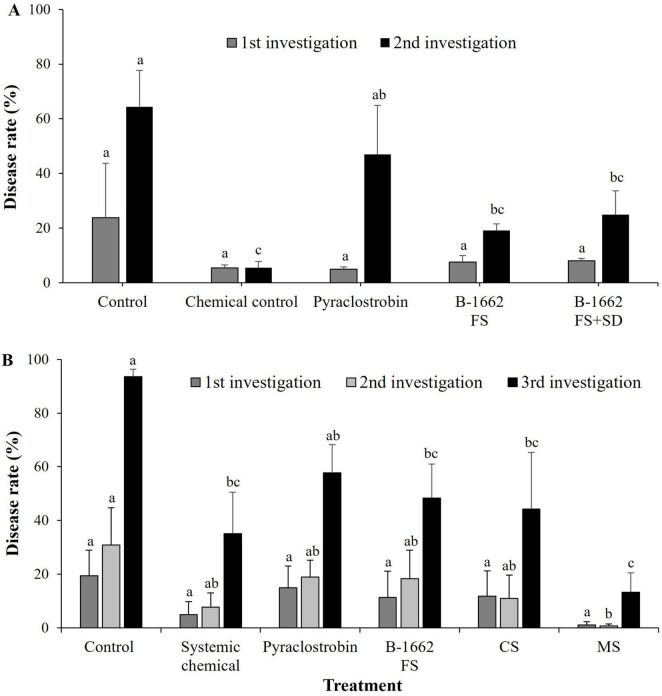
Effect of B-1662 treatment on suppression of anthracnose disease caused by *C. acutatum* under field conditions in 2022 **(A)** and 2023 **(B)**. **(A)** In 2022, after transplanting two-month-old red pepper seedlings in the field, the plants were treated with B-1662 cell suspensions by foliar spray or foliar spray + soil drench or water (control), chemical control, and pyraclostrobin (negative control) for seven times in 60 days. **(B)** In 2023, the plants were treated with B-1662 cell suspensions by foliar spray or cross-spraying or Mixed spraying or water (control), chemical control, and pyraclostrobin (negative control) for seven times in 60 days. Disease rate (%) was recorded from disease-infected red pepper fruits after 10 days of the last treatment, and compared with chemical and non-treated controls. For all the treatments, three different plots with twenty replicates (plants) were used. Differences in letters on bars indicate statistically significant between the treated and the control according to the least significant difference (LSD) (*P* < 0.05). FS, foliar spray, FS+SD: foliar spray+soil drench, CS, Cross spray; MS, Mixed spray.

Bacterium B-1662 was tested for its ability to suppress red pepper anthracnose caused by *C. acutatum* under field conditions for two consecutive years (2022 and 2023). In the first year of the field study, the disease rate (%) of anthracnose in the B-1662 treatment groups has been displayed to show lower when compared to the control group. Especially, in the first investigation, the B-1662 treatment group by FS and FS+SD with cell suspensions of B-1662 treatment group achieved greater disease reduction rates of 8% and 7.5%, respectively. Whereas in the second investigation, the B-1662 treatment group by FS and FS+SD with B-1662 treatment group exhibited disease rates of 19.13% and 25%, respectively (Figure 9A).

On the other hand, in the second year of the field study, during the assessment of disease incidence in the first and second investigation, the disease rates (%) were observed at the same level in the treatments, such as chemical control, pyraclostrobin, B-1662, and CS; while in the treatment of MS the disease rate (%) was drastically reduced (Figure 9B). Whereas in the third investigation, the treatment with MS demonstrated the highest disease reduction with disease rate of 13.2%, while other treatments exhibited disease rates 35%, 57.7%, 48.3%, and 44.2%, in chemical control, pyraclostrobin, B-1662, and CS, respectively. These finding indicates that when B-1662 is applied in combination with chemical pesticides, it exhibits a very high synergistic effect. In two years, B-1662 treatment reduced disease incidence more than pyraclostrobin under field conditions. Overall, treatments with mixed spraying of chemical + B-1662 showed better performance in controlling disease rate (%) compared with the chemical treatments and the untreated control. The slightly higher disease rate (%) has been observed in the FS of B-1662 treatment alone in the second year when compared to the disease rate (%) in the first year; this may be attributed to the higher levels of rainfall in the second year.

## Discussion

Many biological and chemical agents have been used to control bacterial and fungal pathogens under field conditions (Lee et al., 2017), and recently there has been an increasing demand for safe and environmentally friendly agricultural products (Huh and Kim, 2010). As a result, biological control practices are much more in demand as an alternative to synthetic pesticides. Biological control practices are particularly important in organic crop production. Among several kinds of biological control agents (BCAs), *Streptomyces* species are the one, commonly found in soil and are known to have biocontrol capabilities against plant pathogens by producing secondary metabolites with antibacterial and antifungal activity, resulting in high antagonistic capacity against plant pathogens (Gebily et al., 2021). This study describes the characteristics of *S. sporoverrucosus* B-1662 and its biological control effect on the suppression of anthracnose and bitter rot in red pepper and apple, respectively. In this study, *S. sporoverrucosus* B-1662 isolated from freshwater exhibited an *in vitro* antifungal activity against several phytopathogenic fungi, such as *C. acutatum* KACC 42403, *C. coccodes* KACC 48737, and *C. fructicola* CSSG001using a dual culture plate assay with greater inhibition activity when compared to the control.

These results are in consistent with a recent report by Gebily et al. (2021) who stated that *Streptomyces sampsonii* DG1 exhibited a significant inhibitory activity against the mycelial growth of the phytopathogenic fungus *Sclerotinia sclerotiorum*, causes white mold disease in green beans with a reduction of 60.17%, compared with the control. Moreover, Evangelista-Martínez et al. (2020) showed that the antagonistic activity of the *Streptomyces* isolate CACIS-1.5CA was similar to the commercial strain *Streptomyces lydicus* WYEC 108 against several pathogenic fungi. Similarly, Yu et al. (2020) showed that *S. triticiradicis* caused significant inhibitory effects against four phytopathogenic fungi, i.e., *Colletotrichum orbiculare*, *Corynespora cassiicola*, *S. sclerotiorum*, and *Exserohilum turcicum* with an inhibition rate ranging from 48.4 to 69.0%. Further, many reports have demonstrated that *Streptomyces* spp. caused a reduction in mycelial growth of *S. sclerotiorum* due to antifungal substances produced by *Streptomyces* isolates as bioactive compounds and secondary metabolites such as enzymes and antibiotics (Baharlouei et al., 2013). *Streptomyces* spp., are well-known for the tremendous capacity to produce active secondary metabolites, rendering them a significant source of pharmaceutical leads and therapeutic agents. In comparison with the exploitation in pharmaceutical industry, there is only limited application of *Streptomyces* as BCAs in agriculture (Liu et al., 2019). The applications of B-1662 culture filtrate in the present experiments demonstrated its ability to reduce mycelial growths of several fungal phytopathogens due to the secretion of secondary metabolites from B-1662. It could be attributed to the fact that *Streptomyces* spp. produce a variety of secondary metabolites (Ali et al., 2021; Abbasi et al., 2022; Khan et al., 2023), and enzymes such as chitinase and antibiotics (Katarzyna et al., 2018) with extensive use for eco-friendly control of plant pathogens.

In a plate assay, the application of B-1662 cell suspension could alleviate red pepper anthracnose disease caused by *C. acutatum*, and significantly decreased the disease rate by 100% at 10^5^ CFU/mL or 10^7^ CFU/mL. In a previous study, Lee et al. (2012) stated that when *Streptomyces* sp. A1022 applied in a formulated form to control the red pepper anthracnose caused by *Colletotrichum gloeosporioides*, the disease was reduced to greater level when compared to a negative control and the commercial fungicide (azoxystrobin), which implies that *Streptomyces* sp. A1022 SC is very efficient in suppressing the incidence of *C. gloeosporioides*. Similarly, a recent report by Renuka et al. (2023) demonstrated that the results of detached fruit assay revealed that application of liquid bio-formulation of *Stretomyces tuirus* completely inhibited the development of fruit rot symptoms in pepper fruits compared to methanol extracts. In addition, the strain B-1662 exhibited a greater reduction of apple bitter rot disease caused by *C. siamense* than the treatment with CF *in planta* conditions. CF of B-1662 showed significant inhibition against the fungal pathogens. The antifungal compounds might have been present in the CFs. In supportive of our study, a previous report by Prapagdee et al. (2008) stated that CFs of *Streptomyces hygroscopicus* could inhibit *Collelotrichium gloeosporioides* and *Sclerotium rolfsii*. Furthermore, various studies have revealed the biological control potential of different apple endophytic fungi against apple fungal pathogens. For example, Alijani et al. (2016) evaluated the antifungal potential of 15 endophytic species of apple against *Diplodia bulgarica in vitro*. According to Muresan (2017), 55 endophytic isolates of apple among 60 isolates, effectively inhibited the growth of *Venturia inaequalis in vitro*, with *F. oxysporum* FRS09 being the most efficient isolate with 83% suppression effect. On the other hand, Doolotkeldieva and Bobusheva (2017) evaluated the two biological control agents of *Trichoderma viride* Pers. and *Streptomyces* sp. against apple scab disease, and the application of *Streptomyces* sp. isolates were less efficient than the *T. viride* in disease suppression.

We tested B-1662 for its ability to produce indole-3-acetic acid (IAA), which is associated with its ability to enhance plant development, and found that B-1662 cell suspensions produced IAA. IAA is a popular auxin-based plant growth regulator. It plays a key role in plant growth and development by inducing cell elongation and division (Fu et al., 2015). The ability of bacteria to produce IAA is an important characteristic that can influence plant growth. IAA enhances root growth by increasing root length and surface area, allowing plants to utilize more nutrients from the soil (Peng et al., 2023). The strain *Streptomyces fradiae* NKZ-259 demonstrated IAA production that promoted tomato growth by exhibiting the highest root and shoot lengths, fresh and dry weights, and root and shoot diameters (Myo et al., 2019). The IAA-producing activity of PGPR varies among species and is greatly influenced by culture conditions, growth stage and substrate ability (Mirza et al., 2001). Manulis et al. (1994) studied the production of IAA and the pathways of its synthesis by various *Streptomyces* spp., including *Streptomyces violaceus*, *S. griseus*, *S. exfoliates*, *S. coelicolor*, and *S. lividans*. This finding is consistent with the knowledge that actinomycetes possess the ability to produce IAA in the presence of a suitable precursor such as L-tryptophan (El-Tarabily and Sivasithamparam, 2006). However, other pathways may be included in this mechanism because some bacteria possess more than one pathway (Spaepen et al., 2007). Reddy et al. (2016) isolated *Streptomyces atrovirens* from groundnut roots. This bacterium has shown excellent growth-promoting activity not only on groundnut but also on a number of other crops.

Furthermore, treatment with B-1662 cell suspensions inhibited conidia germination when mixed with suspensions of fungal pathogen conidia, greatly impeded conidia formation in comparison to the untreated control group. *Streptomyces* spp. are the most capable genus of capable of producing bioactive compounds that are effective against a wide variety of pathogens, according to accumulating evidence (Newman and Cragg, 2016). Recently, Zhou et al. (2022) demonstrated that the *Streptomyces* sp. HSL-9B strain and its extracts exhibited potent inhibitory effects on developing pathogenic mycelia and conidia of *Colletotrichum gloeosporioides*. Consequently, the growth of mango anthracnose was effectively suppressed, achieving a control efficacy of 79.7%. On the other hand, specific isolates of *Bacillus*, such as *Brevibacillus halotolerans* B-4359, exhibited no inhibitory effect on conidial germination when combined with conidial suspensions of the fungus (Kim et al., 2023). The results presented here align with those of a previous investigation (Besset-Manzoni et al., 2019), which observed that BCAs demonstrated beneficial effectiveness *in planta* but did not manifest any antagonistic effects *in vitro*. Shu et al. (2017) found that cell-free CFs from *Streptomyces katrae* strain NB20 inhibited mycelial growth and conidial germination of *Colletotrichum musae* by 97.7 ± 0.9% and 95.0 ± 0.6%, respectively. A recent study by Kim et al. (2019) found that the application of secondary metabolites derived from *Streptomyces rectiviolaceus* DY46 reduced the prevalence of gray mold in tomatoes treated with CF by 80% compared to the control group. Furthermore, the CF of *Streptomyces* sp. CB-75 significantly inhibited spore germination of *C. musae* and *C. gloeosporioides*, as demonstrated by contraction, collapse, and tortuosity morphology (Chen et al., 2018).

The application of B-1662 in the field demonstrated an outstanding biological control effect in both years 2022 and 2023, with a slightly greater effect in the first year compared to the second year. This might be due to weather changes in the second year. A preventive treatment with *Streptomyces* sp. H4 strain considerably reduced the severity of anthracnose disease while preserving fruit firmness and color on harvested strawberry fruits (Li et al., 2021). On the contrary, the *Brevibacillus halotolerans* B-4359 strain failed to exhibit direct antagonistic activity against the pathogen *in vitro* or inhibits spore germination. However, it did induce the expression of actin (ACT), a housekeeping gene, and CaPR1, a marker gene for systemic resistance in plants (Kim et al., 2023). An assortment of parameters influence disease incidence in the field, including environmental factors, soil microorganisms, and the characteristics of the pathogen (Wang et al., 2019; Li et al., 2023). Our findings are consistent with those of a previous study by Lee et al. (2012), which found *Streptomyces* sp. A1022 SC provided superior growth conditions and greater protection against anthracnose for pepper plants compared to the untreated control and the commercial fungicide azoxystrobin.

Furthermore, in this study, the antifungal volatile organic compounds (VOCs) of B-1662 showed antifungal effects against *C. siamense*, and the antifungal VOCs of B-1662 were identified by GC-MS. As a result, butane, 1,1-dibutoxy- substance was detected in the butanol fraction, which was associated with antifungal activity. In addition, the antifungal activity was also found to have significant thermal and light stability, which suggested that it was related to the high G+C content of B-1662. VOCs produced by microorganisms may prevent postharvest rot in fruits. Recently, a study (Jepsen et al., 2022) has examined whether VOCs from different species of *Streptomyces* can control infection in apples caused by the fungal pathogen *Colletotrichum acutatum*.

Our study further reports the whole-genome sequence of strain B-1662, which consists of 8,254,451 bp on the chromosomes. The genome of the B-1662 strain was compared with those of similar species within the same genus using a whole-genome analysis. The non-ribosomal peptides (NRPs) refer to peptide compounds that assemble natural or non-natural amino acids or modify amino acids through modular non-ribosomal peptide synthetases (Maruyama et al., 2012). The most prevalent BGCs were those associated with polyketide synthases (PKS) and NRPS; these two BGCs synthesize active substances of macrolides and non-ribosomal peptides, respectively (Theobald et al., 2019). The observed outcomes validated the profusion of naturally active substances derived from *Streptomyces*, a finding that could potentially contribute to the advancement of antibiotic development. In particular, all *Streptomyces* were found to contain ectoine and siderophore BGCs (Wang et al., 2021). Polyketide compounds, which possess a wide range of biological activities such as anti-pathogenic properties, are among the most structurally and functionally diverse natural products (Miyanaga, 2017). NRPS finds extensive application in both the agricultural and medicine sectors, functioning as an antifungal, immunosuppressant, lipid-lowering agent (Maansson et al., 2016). A total of 27 secondary metabolite gene clusters were identified in the B-1662 strain genome through anti-SMASH analysis. Among these clusters, the genes encoding desferrioxamine B, a siderophore, avermitilol, a compound whose activity remains unknown, and SapB, a substance implicated in hyphae formation, exhibited a similarity of 100%. The metabolites of *Streptomyces* isolated from the soil also contain large amounts of terpenes (Wang et al., 2013). Terpenoids have been displayed to exhibit a potent antibacterial effect against food-borne pathogens (Gallucci et al., 2009). This finding supports our results, which demonstrated that the strain B-1662 produces terpenes with antifungal effect via BGCs. Analysis of the BGCs distribution among several strains of *Streptomyces lunelactis* revealed that some clusters are found exclusively in a few or a single strain, while others are extensively spread (Martinet et al., 2020). Similarly, two reports by Komaki et al. (2018, 2020) showed the diversity of NRPS and PKS in *Streptomyces*, which are taxonomically closely related. Numerous clusters of genes encoded type-a polyketide synthases (t1pks) and non-ribosomal peptides (NRP), which are the most common prevalent enzymes involved in the biosynthesis of diverse secondary metabolites. This suggests that these enzymes may serve a potential function as plant interaction molecule or biocontrol agents (Li et al., 2015; Chevrette et al., 2019).

## Conclusion

In conclusion, the *S. sporoverrucosus* B-1662 isolate procured from FBCC has showed remarkable antagonistic activity against fungal pathogens, such as *C. acutatum* causing anthracnose in red pepper, and *C. siamense* causing bitter rot in apples. B-1662 cultures exhibited a significant inhibitory effect on red pepper anthracnose and conidial germination *in vitro*. The strain B-1662 produced IAA. Field trials conducted in Andong, Gyeongbuk Province, Korea demonstrated that B-1662 treatment effectively controlled red pepper anthracnose. The isolate B-1662 was identified as *S. sporoverrucosus* based on whole-genome sequencing. Whole-genome sequencing of B-1662 revealed antimicrobial secondary metabolites including desferrioxamine B, avermitilol, and SapB. Based on the findings, B-1662 appeared to be a potentially effective biocontrol agent for environmentally sustainable management of plant pathogens. Future research will employ proteomic and transcriptome techniques to investigate the signaling pathways implicated in the antagonistic effects of secondary metabolites.

## Data Availability

The datasets presented in this study can be found in online repositories. The names of the repository/repositories and accession number(s) can be found in the article/Supplementary material.

## Footnotes

1
http://eggnogdb.embl.de

2
https://dl.secondarymetabolites.org/releases/7.0.0/
