# Supplementary material for: Evaluation of Streptomyces sporoverrucosus B-1662 for biological control of red pepper anthracnose and apple bitter rot diseases in Korea
Source: Front Microbiol. 2024 Nov 28;15:1429646. doi: 10.3389/fmicb.2024.1429646 (PMC11634798; doi:10.3389/fmicb.2024.1429646)
Supplement: Supplementary file 1 [file Table_1.docx]

**Supplementary information**

**Evaluation of *Streptomyces* strains for biological control of red pepper anthracnose and apple bitter rot diseases in Korea**

DaYoung Kim^1^, Jungyeon Kim^1^, Younmi Lee^1^, Kotnala Balaraju^2^, Ye-Ji Hwang^3^, Mi-Hwa Lee^4^, Wonsu Cheon^5^**,** Hye Yeon Mun^5^, Chang Soo Lee^5^, and Yongho Jeon^1*^

^1^Department of Plant Medicals, Andong National University, Andong, Republic of Korea,

^2^Agricultural Science and Technology Research Institute, Andong National University, Andong, Republic of Korea,

^3^Using Technology Development Department, ^4^Diversity Conservation Research Department, ^5^Biological Resources Research Department, Nakdonggang National Institute of Biological Resources, Sangju, Republic of Korea

*Corresponding author: Prof. Yongho Jeon

E-mail: yongbac@andong.ac.kr

Tel: +82-54-820-5507,

Fax: +82-54-820-6320

**Running title:** Characterization of *Streptomyces* strains

**Supplementary Tables**

**Supplementary Table 1. Treatment schedule and details of the field trial in the year 2022**

| Day After Transplaantation (DAT) /  Treatment | 49  (24, June) | 61  (6, July) | 70  (15, July) | 81  (26, July) | 91  (5, August) | 102  (16, August) |
| --- | --- | --- | --- | --- | --- | --- |
| Control | - | - | - | - | - | - |
| Chemical control | Acibenzolar-s methyl  (1%) + mancozeb  (48%) | Propineb (70%) | Pyraclostrobin  (22.9%) | Chlorothalonil  (20%) + Difenoconazole  (4%) | Dithianon  (24%) +  Pyraclostrobin  (8%) | Prochloraz  (25%) +  Tebuconazole  (12.5%) |
| Pyraclostrobin | Pyraclostrobin  (22.9%) | Pyraclostrobin  (22.9%) | Pyraclostrobin  (22.9%) | Pyraclostrobin  (22.9%) | Pyraclostrobin  (22.9%) | Pyraclostrobin  (22.9%) |
| B-1662  Foliar spray | B-1662 | B-1662 | B-1662 | B-1662 | B-1662 | B-1662 |
| B-1662  Foliar spray and  Soil drenching | B-1662 | B-1662 | B-1662 | B-1662 | B-1662 | B-1662 |

^a^; 10g/20l

**Supplementary Table 2. Treatment schedule and details of the field trial in the year 2023**

| Day After Transplaantation (DAT) /  Treatment | 63  (7, July) | 76  (20, July) | 87  (31, July) | 98  (11, August) | 108  (21, August) |
| --- | --- | --- | --- | --- | --- |
| Control | - | - | - | - | - |
| Chemical control | Acibenzolar-s methyl  (1%) + mancozeb  (48%) | Propineb (70%) | Pyraclostrobin  (22.9%) | Chlorothalonil  (20%) + Difenoconazole  (4%) | Dithianon  (24%) +  Pyraclostrobin  (8%) |
| Pyraclostrobin | Pyraclostrobin  (22.9%) | Pyraclostrobin  (22.9%) | Pyraclostrobin  (22.9%) | Pyraclostrobin  (22.9%) | Pyraclostrobin  (22.9%) |
| B-1662  Foliar spray | B-1662 | B-1662 | B-1662 | B-1662 | B-1662 |
| Cross-spray | B-1662 | Propineb (70%) | B-1662 | Chlorothalonil  (20%) + Difenoconazole  (4%) | B-1662 |
| Mix-spray | B-1662 +  Acibenzolar-s methyl  (1%) + mancozeb  (48%) | B-1662 +  Propineb (70%) | B-1662 +  Pyraclostrobin  (22.9%) | B-1662 +  Chlorothalonil  (20%) + Difenoconazole  (4%) | B-1662 +  Dithianon  (24%) +  Pyraclostrobin  (8%) |

**Supplementary Table 3.** Information of Chemical fungicides used in field experiments.

| Name | Molecular  Ratio (%) | Formulation | Concentration in field |
| --- | --- | --- | --- |
| Acibenzolar-S-Methyl +  Mancozeb | 1 + 48 | Wettable powder | 1㎎/㎖ |
| Propineb | 70 | Wettable powder | 2㎎/㎖ |
| Pyraclostrobin | 22.9 | Emulsifiable concentrate | 0.25 ㎕/㎖ |
| Chlorothalonil +  Difenoconazole | 20 + 4 | Suspension concentrate | 1 ㎕/㎖ |
| Dithianon +  Pyraclostrobin | 24 + 8 | Suspo mulsion | 0.5 ㎕/㎖ |
| Prochloraz +  Tebuconazole | 25 + 12.5 | Emulsifiable concentrate | 0.5 ㎕/㎖ |

**Supplementary Table 4**. Cultural characteristics of *Streptomyces sporoverrucosus* B-1662 on various ISP media

| **Medium** | **Cultural characteristics of strain B-1662** | | |
| --- | --- | --- | --- |
|  | **Growth of the mycelim** | **Mycelia color** | **Soluble pigments** |
| Trypton yeast extract agar (ISP1) | Moderate | white to yellow | +ve |
| Yeast extract-malt extract agar (ISP2) | Poor | white to yellow | +ve |
| Oatmeal agar (ISP3) | Good | red-brownish white | +ve |
| Inorganic salt starch agar (ISP4) | Moderate | light camel brown | -ve |
| Glycerol asparagine agar (ISP5) | Poor | white | -ve |

**Supplementary Table 5**. Bioactive Compounds detected in the n-Butanol 1 layer extract of *Streptomyces sporoverrucosus* B-1662

| **Compound name** | **Molecular formula** | **Retention Time (min)** | **Peak area (%)** |
| --- | --- | --- | --- |
| Octane, 4-methyl- | C1_2_H_2_6O_2_ | 5.283 | 0.9 |
| Cyclohexanone | C_6_H_10_O | 5.997 | 0.6 |
| 2-Pyrrolidinone, 1-methyl- | C_5_H_9_NO | 8.672 | 10.8 |
| Piperidine, 4-methyl- | C_6_H_13_N | 8.754 | 6.9 |
| Butane, 1,1-dibutoxy- | C_12_H_26_O_2_ | 12.32 | 40.0 |
| Benzaldehyde, 4-propyl- | C_10_H_12_O | 12.91 | 2.5 |
| Phenol, 2,4-bis(1,1-dimethylethyl)- | C_17_H_30_OSi | 17.538 | 9.3 |
| 9-Octadecenamide, (Z)- | C_18_H_35_NO | 30.915 | 0.9 |
| 9-Octadecenamide, (Z)- | C_18_H_35_NO | 34.015 | 26.1 |
| Octadecanamide | C_18_H_37_NO | 34.44 | 2.0 |

**Supplementary Table 6.** Effect of temperature, light treatments on the antifungal activity of culture filtrates of *Streptomyces sporoverrucosus* B-1662

| **Treatment** | ***Colletotrichum siamense CGCP6*** | |
| --- | --- | --- |
|  | **Mycelial length (mm)** | **Inhibition rate (%)** |
| Control | 84.6±0.3 | 0 |
| Culture Filtrate | 39.6±0.3 | 53.1±0.3 |
| Heat temperature treatment |  |  |
| 37℃, 1 h | 38±0.5 | 55.1±0.6 |
| 50℃, 1 h | 49.3±0.6 | 41.7±0.7 |
| 70℃, 1 h | 55±2.0 | 35±2.4 |
| 100℃, 1 h | 57.6±0.3 | 31.8±0.3 |
| 121℃, 20 min | 58.3±0.8 | 31.1±1.0 |
| Low temperature treatment |  |  |
| -20℃, 1 h | 40 | 52.7 |
| Photostability |  |  |
| UV light, 1 h | 41±0.5 | 51.5±0.6 |

**Supplementary Table 7.** Result of assembly: 4 contigs were formed

| **Contig name** | **Length (bp)** | **GC (%)** | **CDS** | **tRNA** | **rRNA** |
| --- | --- | --- | --- | --- | --- |
| Contig 1 | 7,457,343 | 72.5 | 6,474 | 87 | 21 |
| Contig 2 | 532,210 | 71.3 | 513 | 3 | 0 |
| Contig 3 | 157,211 | 72.1 | 148 | 0 | 0 |
| Contig 4 | 107,687 | 69.5 | 127 | 0 | 0 |
| Total | 8,254,451 | 72.4 | 7,262 | 90 | 21 |
