# Supplementary material for: Evaluation of Streptomyces sporoverrucosus B-1662 for biological control of red pepper anthracnose and apple bitter rot diseases in Korea
Source: Front Microbiol. 2024 Nov 28;15:1429646. doi: 10.3389/fmicb.2024.1429646 (PMC11634798; doi:10.3389/fmicb.2024.1429646)
Supplement: Supplementary file 2 [file Presentation_1.pptx]

## Slide 1
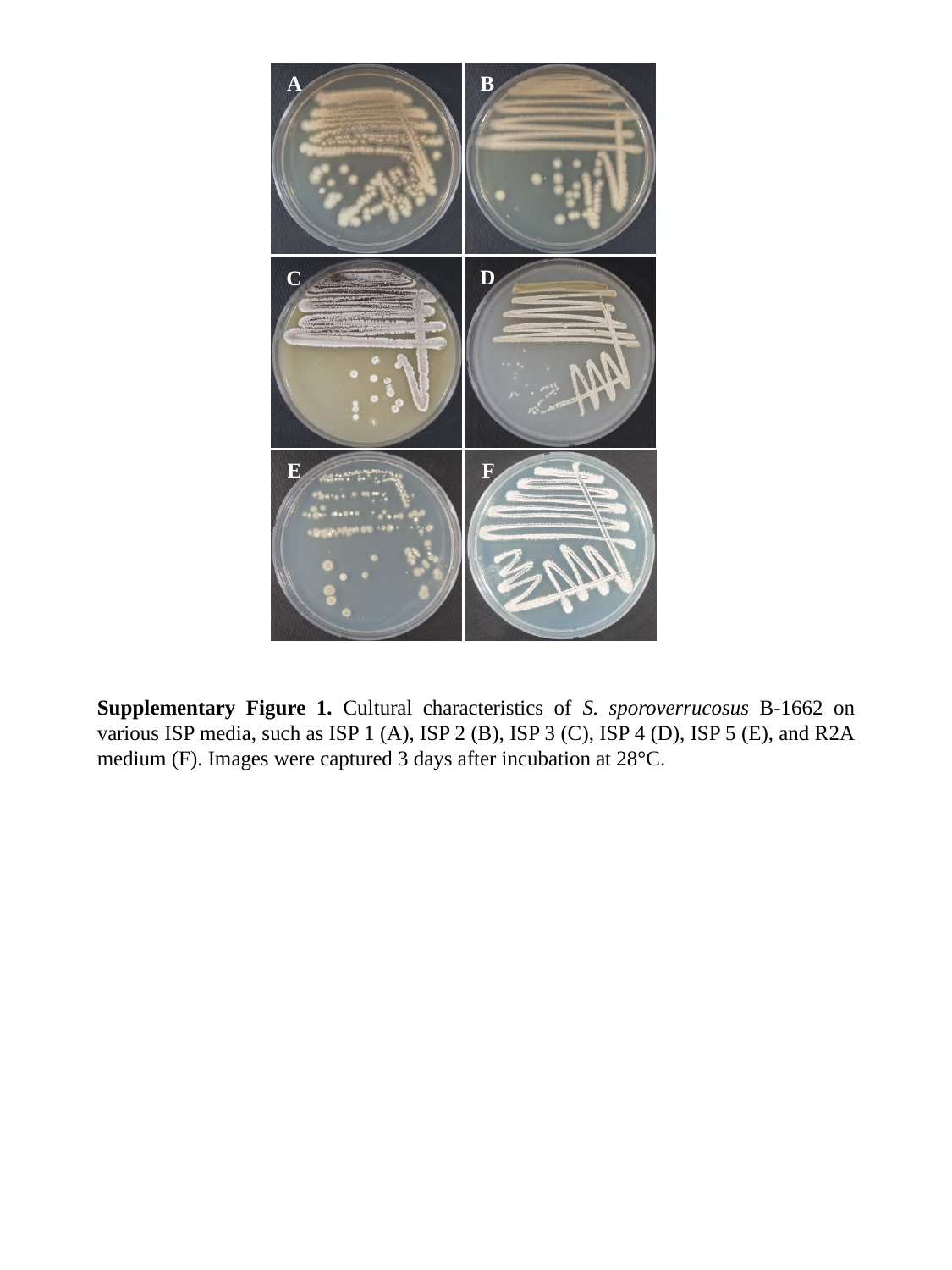

A
B
D
C
F
E
Supplementary Figure 1. Cultural characteristics of S. sporoverrucosus B-1662 on various ISP media, such as ISP 1 (A), ISP 2 (B), ISP 3 (C), ISP 4 (D), ISP 5 (E), and R2A medium (F). Images were captured 3 days after incubation at 28°C.

## Slide 2
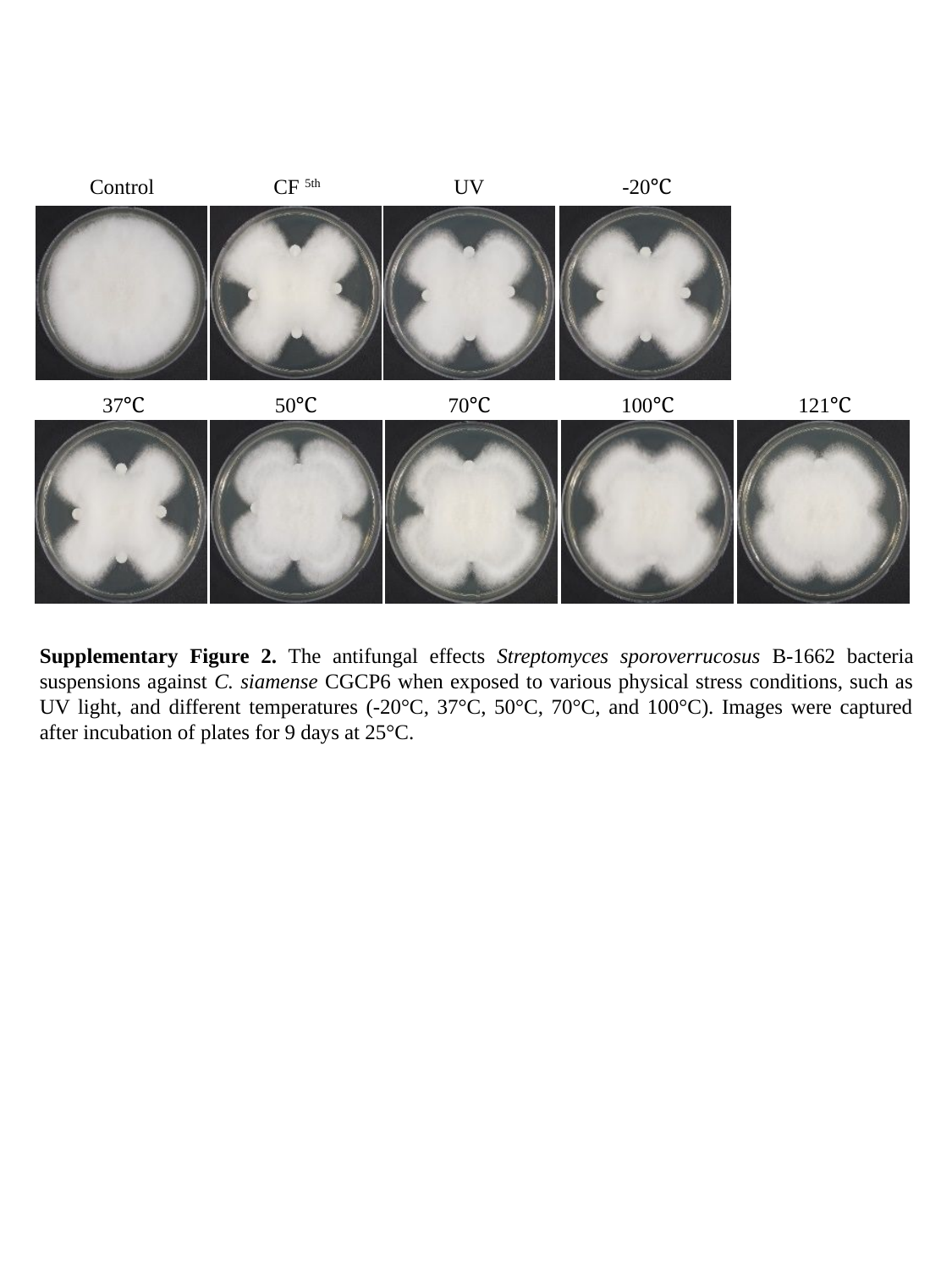

-20℃
CF 5th
UV
Control
37℃
50℃
70℃
100℃
121℃
Supplementary Figure 2. The antifungal effects Streptomyces sporoverrucosus B-1662 bacteria suspensions against C. siamense CGCP6 when exposed to various physical stress conditions, such as UV light, and different temperatures (-20°C, 37°C, 50°C, 70°C, and 100°C). Images were captured after incubation of plates for 9 days at 25°C.

## Slide 3
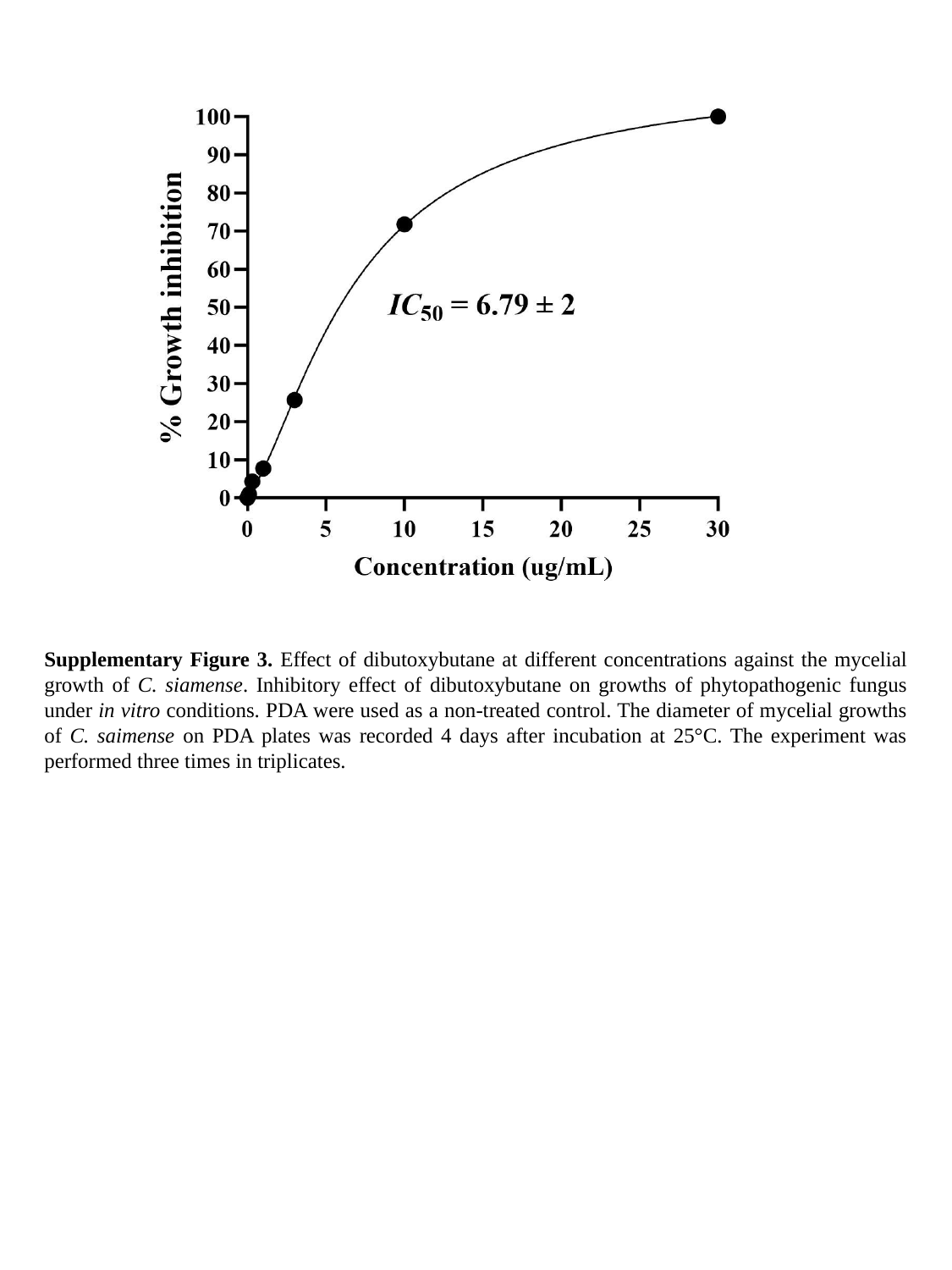

Supplementary Figure 3. Effect of dibutoxybutane at different concentrations against the mycelial growth of C. siamense. Inhibitory effect of dibutoxybutane on growths of phytopathogenic fungus under in vitro conditions. PDA were used as a non-treated control. The diameter of mycelial growths of C. saimense on PDA plates was recorded 4 days after incubation at 25°C. The experiment was performed three times in triplicates.

## Slide 4
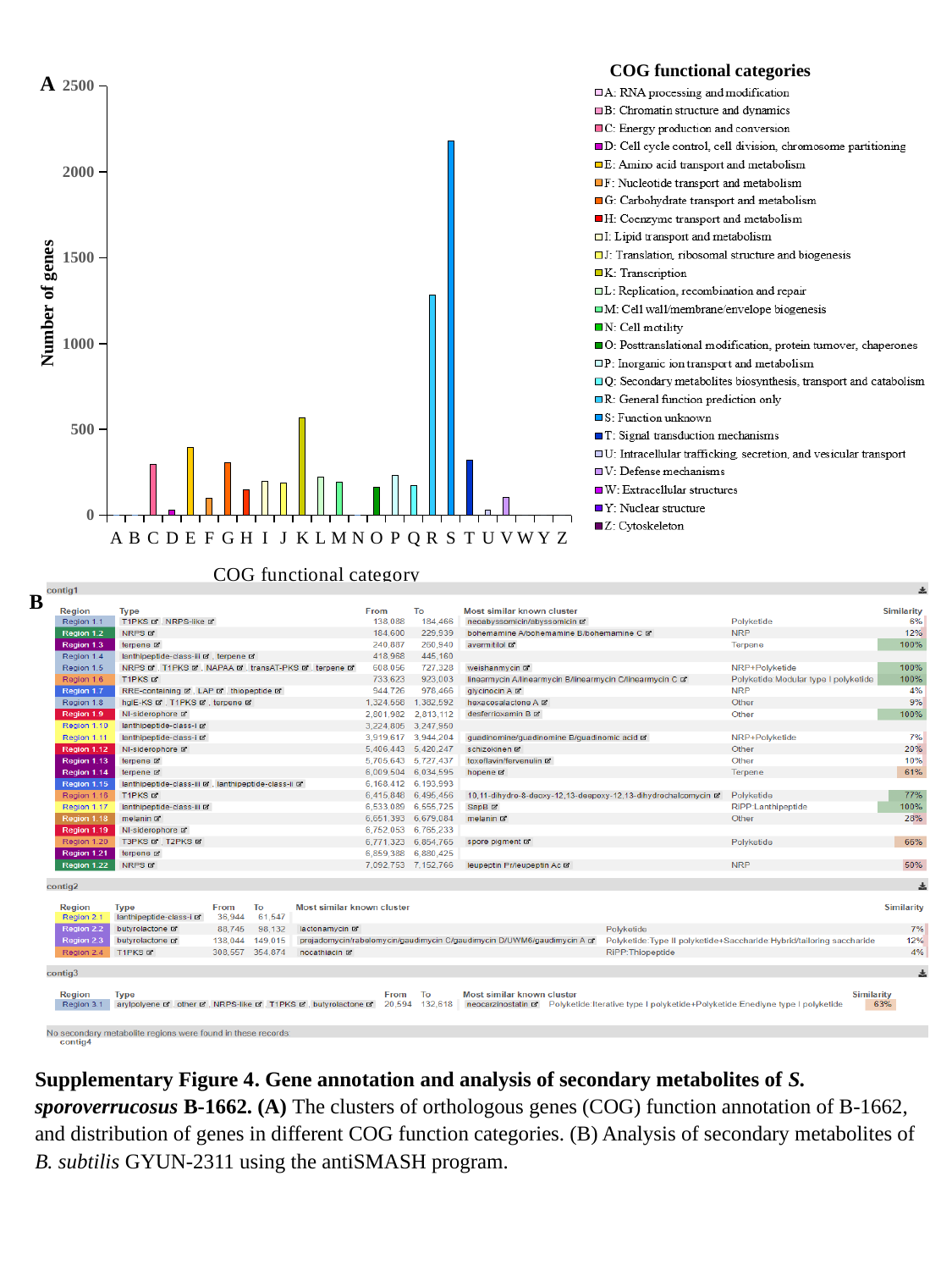

### Chart
| Category | |
|---|---|
| A | 1.0 |
| B | 1.0 |
| C | 294.0 |
| D | 30.0 |
| E | 394.0 |
| F | 98.0 |
| G | 308.0 |
| H | 147.0 |
| I | 200.0 |
| J | 189.0 |
| K | 566.0 |
| L | 224.0 |
| M | 193.0 |
| N | 1.0 |
| O | 163.0 |
| P | 232.0 |
| Q | 172.0 |
| R | 1284.0 |
| S | 2179.0 |
| T | 320.0 |
| U | 31.0 |
| V | 103.0 |
| W | 0.0 |
| Y | 0.0 |
| Z | 0.0 |COG functional categories
A
Number of genes
B
Supplementary Figure 4. Gene annotation and analysis of secondary metabolites of S. sporoverrucosus B-1662. (A) The clusters of orthologous genes (COG) function annotation of B-1662, and distribution of genes in different COG function categories. (B) Analysis of secondary metabolites of B. subtilis GYUN-2311 using the antiSMASH program.

## Slide 5
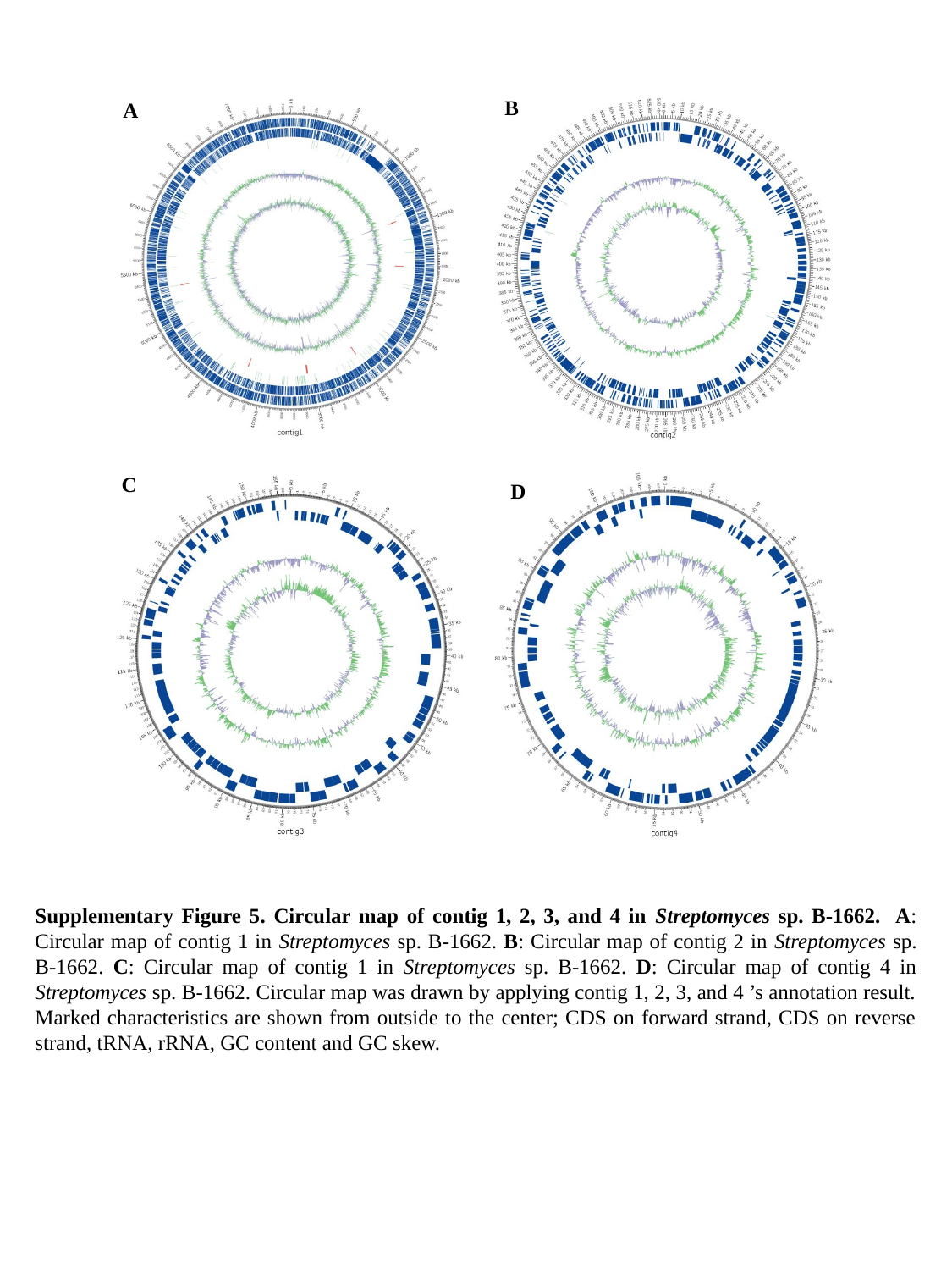

B
A
C
D
Supplementary Figure 5. Circular map of contig 1, 2, 3, and 4 in Streptomyces sp. B-1662. A: Circular map of contig 1 in Streptomyces sp. B-1662. B: Circular map of contig 2 in Streptomyces sp. B-1662. C: Circular map of contig 1 in Streptomyces sp. B-1662. D: Circular map of contig 4 in Streptomyces sp. B-1662. Circular map was drawn by applying contig 1, 2, 3, and 4 ’s annotation result. Marked characteristics are shown from outside to the center; CDS on forward strand, CDS on reverse strand, tRNA, rRNA, GC content and GC skew.
